# Pre-Exposure Prophylaxis Integration Into Family Planning Services at Title X Clinics in the Southeastern United States: Protocol for a Mixed Methods Hybrid Type I Effectiveness Implementation Study (Phase 2 ATN 155)

**DOI:** 10.2196/18784

**Published:** 2020-09-25

**Authors:** Anandi N Sheth, Sophia A Hussen, Cam Escoffery, Lisa B Haddad, Leah Powell, Nakita Brown, Teresa R Filipowicz, Micah McCumber, Maria Sanchez, Laura Renshaw, Matthew A Psioda, Jessica M Sales

**Affiliations:** 1 Department of Medicine, Division of Infectious Diseases School of Medicine Emory University Atlanta, GA United States; 2 Hubert Department of Global Health Rollins School of Public Health Emory University Atlanta, GA United States; 3 Department of Behavioral Sciences and Health Education Rollins School of Public Health Emory University Atlanta, GA United States; 4 Department of Gynecology and Obstetrics School of Medicine Emory University Atlanta, GA United States; 5 Department of Biostatistics Collaborative Studies Coordinating Center University of North Carolina at Chapel Hill Chapel Hill, NC United States

**Keywords:** HIV, pre-exposure prophylaxis, implementation science, family planning services

## Abstract

**Background:**

Adolescent and young adult women (AYAW), particularly racial and ethnic minorities, in the Southern United States are disproportionately affected by HIV. Pre-exposure prophylaxis (PrEP) is an effective, scalable, individual-controlled HIV prevention strategy that is grossly underutilized among women of all ages and requires innovative delivery approaches to optimize its benefit. Anchoring PrEP delivery to family planning (FP) services that AYAW already trust, access routinely, and deem useful for their sexual health may offer an ideal opportunity to reach women at risk for HIV and to enhance their PrEP uptake and adherence. However, PrEP has not been widely integrated into FP services, including Title X–funded FP clinics that provide safety net sources of care for AYAW. To overcome potential implementation challenges for AYAW, Title X clinics in the Southern United States are uniquely positioned to be focal sites for conceptually informed and thoroughly evaluated PrEP implementation science studies.

**Objective:**

The objective of this study is two-fold: to evaluate multilevel factors associated with the level of PrEP adoption and implementation (eg, PrEP screening, counseling, and prescription) within and across 3 FP clinics and to evaluate PrEP uptake, persistence, and adherence among female patients in these clinics over a 6-month follow-up period.

**Methods:**

Phase 2 of Planning4PrEP (Adolescent Medicine Trials Network for HIV/AIDS Interventions 155) is a mixed methods hybrid type 1 effectiveness implementation study to be conducted in three clinics in Metro Atlanta, Georgia, United States. Guided by the Exploration, Preparation, Implementation, and Sustainment framework, this study will prepare clinics for PrEP integration via clinic-wide trainings and technical assistance and will develop clinic-specific PrEP implementation plans. We will monitor and evaluate PrEP implementation as well as female patient PrEP uptake, persistence, and adherence over a 6-month follow-up period.

**Results:**

Phase 2 of Planning4PrEP research activities began in February 2018 and are ongoing. Qualitative data analysis is scheduled to begin in Fall 2020.

**Conclusions:**

This study seeks to evaluate factors associated with the level of PrEP adoption and implementation (eg, PrEP screening, counseling, and prescription) within and across 3 FP clinics following training and implementation planning and to evaluate PrEP uptake, persistence, and adherence among female patients over a 6-month follow-up period. This will guide future strategies to support PrEP integration in Title X–funded clinics across the Southern United States.

**Trial Registration:**

ClinicalTrials.gov NCT04097834; https://clinicaltrials.gov/ct2/show/NCT04097834

**International Registered Report Identifier (IRRID):**

DERR1-10.2196/18784

## Introduction

Adolescent and young adult women (AYAW) in the Southern United States are disproportionately affected by HIV in comparison with AYAW residing in other regions [1]. Southern states account for nearly half of the new HIV diagnoses, despite having only 37% of the nation’s population [2]. In Atlanta, 4 metro counties (Cobb, DeKalb, Fulton, and Gwinnett) were identified as critical HIV hotspots in the Health and Human Services’ *Ending the HIV Epidemic: A Plan for America* [3]. Reducing HIV among women in the Southern United States in general, and Metro Atlanta specifically, is a priority.

Since approval of daily oral PrEP as an effective HIV prevention strategy [4], there has been wide-scale endorsement to bring PrEP to scale through dissemination and implementation efforts in the United States [5-7], but PrEP remains underutilized in women relative to need [8-10]. The first steps in PrEP adoption are ensuring that those who can benefit from PrEP are aware of it and ensuring PrEP is accessible in health care settings where they seek care [11,12]. However, there is low knowledge of PrEP among US women [13-18] and women’s health providers [19], although studies suggest that family planning (FP) providers are willing to prescribe PrEP once trained [19], and women are interested in taking PrEP once informed [20,21].

Anchoring PrEP delivery to health services such as FP clinics that AYAW already trust, access routinely, and deem useful for their sexual health is of great appeal. FP providers in areas with high HIV incidence are ideal potential PrEP providers because most (60%) AYAW utilize and trust FP providers for sexual health and preventative services [22]. Many are also important safety net sources of care for AYAW, particularly in states that did not expand Medicaid and are expected to offer HIV prevention services as part of quality FP [23].

However, PrEP has not been widely integrated into FP services in the United States. Our 2018 survey among individuals working in Title X clinics across the Southern United States revealed that the majority (approximately 80%) reported working in clinics that did not provide PrEP [24]. Our findings are in line with a 2015 national survey of FP providers in the United States, which reported low PrEP knowledge and use, especially in the South [19]. In addition, women have lower PrEP uptake and persistence than men [25], highlighting that significant implementation challenges exist among women.

Very few models exist that describe the organizational processes and strategies associated with successful integration of PrEP delivery in new clinic settings and none, to our knowledge, exists for FP clinics, including those supported by Title X funding. Despite the presence of clinical trial efficacy data for PrEP among both men and women, *real-world* effectiveness data on PrEP among women have only become recently available from studies among women in sub-Saharan Africa [26], thus lagging behind PrEP effectiveness research in men. Effectiveness-implementation hybrid designs are innovative implementation science approaches for more rapid *research to practice* translations. The hybrid type 1 design is a blended design that is ideally suited to test the effects of a clinical intervention on relevant patient outcomes in *real-world* settings (eg, PrEP uptake, adherence, and persistence among women) while gathering information on its implementation [27]. Given the aforementioned gaps in the literature on both effectiveness and implementation of PrEP among women in the United States, research employing a hybrid type 1 design is ideal for advancing the knowledge in this critical but understudied area and may inform future interventions to optimize PrEP delivery in this setting. Finally, maximizing the potential benefits of PrEP requires understanding the key provider- and patient-related steps in integrated PrEP delivery. These steps comprise the PrEP engagement cascade and have been proposed as a conceptual framework to identify and understand the key steps needed to maximize the benefits of PrEP [28-30]. However, there is a dearth of research exploring factors associated with these provider- and patient-related steps among women utilizing FP clinics in the Southeastern United States.

The Adolescent Medicine Trials Network for HIV/AIDS Interventions (ATN) is a research program that aims to defeat the rising HIV epidemic among adolescents and young adults in the United States. The overarching goal of the ATN is to increase awareness of HIV status in youth and, for those diagnosed with HIV, increase access to health care. The ATN develops and conducts behavioral, community-based, translational, therapeutic, microbicide, and vaccine trials in youth who are at risk for or living with HIV, with a focus on the inclusion of minors. Our study is funded as part of the ATN (ATN 155). The combined findings and resulting tools and trainings will be valuable for PrEP integration in Title X–funded or similarly structured FP clinics and to inform future interventions to optimize PrEP delivery for AYAW. Phase 2 of Planning4PrEP was directly guided by phase 1 of the study; review phase 1 protocol paper for complete details [31]. In this paper, we describe only the research protocol for the phase 2 study. The objective of this study is to evaluate multilevel factors associated with the level of PrEP adoption and implementation within and across 3 FP clinics and to evaluate PrEP uptake, persistence, and adherence among female patients in these clinics over a 6-month follow-up period.

## Methods

### Study Design

This study is a mixed methods hybrid type 1 effectiveness implementation study with two key objectives. Objective one is to evaluate factors associated with clinic-level PrEP adoption and implementation at three FP clinics. This objective will be measured both quantitatively and qualitatively. Quantitative data include clinic-level aggregate data obtained via chart abstraction, staff-level data obtained via a web-based survey, and patient-level data collected via interviewer-assisted exit surveys. In addition to the web-based survey, staff participants will also take part in key informant interviews and focus groups. Objective two is to evaluate the effect of PrEP integration on PrEP uptake, persistence, and adherence among female patient participants over a 6-month period. This objective will be measured via a prospective cohort study of 300 PrEP-eligible women across all three clinics. To characterize each step in the PrEP cascade, within this cohort, we aim to recruit women who may benefit from PrEP (as determined via HIV risk assessment by the patient or provider), regardless of whether PrEP was prescribed.

**Figure 1:**
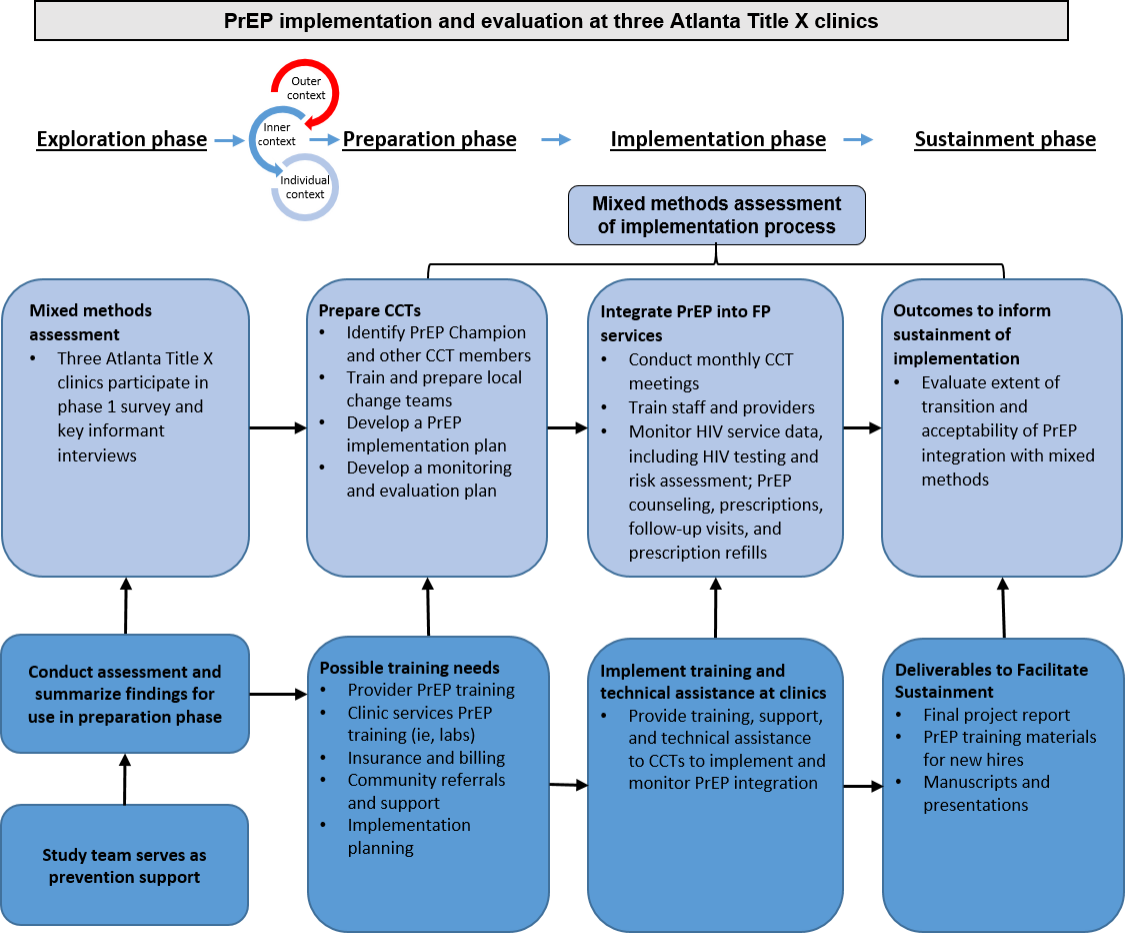
Pre-exposure prophylaxis implementation and evaluation process at three Atlanta Title X clinics including a federally qualified health center clinic, specialized family planning clinic, and hospital-based family planning clinic. CCT: Clinic Change Team; FP: family planning; PrEP: pre-exposure prophylaxis.

Both objectives directly correlate to different stages of the Exploration, Preparation, Implementation, and Sustainment (EPIS) framework (Figure 1). Methods pertinent to objective one fall under preparation, implementation and sustainment phases, whereas methods pertinent to objective two fall under the implementation and sustainment phases. Importantly, objective two methods are essential for better understanding real-world PrEP effectiveness among women in the United States. Secondary outcome measures among staff participants will be mapped to the Consolidated Framework for Implementation Research (CFIR) framework.

### Theoretical Frameworks

#### EPIS Framework

The EPIS framework [32] is a phased, multilevel (ie, clinic and provider and staff-level) approach to conceptualizing implementation research that provides both structure and a process to implementation across 4 distinct stages. *Exploration*, the first stage, was assessed in the first phase of this study [31]. The next step, *preparation*, involves bringing together relevant stakeholders in a planning process to support effective implementation and includes developing a proposed plan to address barriers (eg, through clinic-wide PrEP trainings and capacity building with local PrEP technical assistance providers). The *implementation* stage begins after training or when other system changes are required for the PrEP end. When implementation reaches normalized operations (eg, a clinic can onboard a new provider and ensure that they are prepared to screen and provide PrEP), *sustainment* begins. This study will thoroughly evaluate EPIS stages from *preparation* to the early *sustainment* stage.

#### CFIR

Using the CFIR framework [33], we will assess which inner and outer contextual factors (barriers and facilitators) influence the level of adoption of PrEP services in Title X clinics serving women in the Southern United States. The CFIR provides a menu of constructs that have been identified as important for implementation success [33]. The CFIR captures the complex, multilevel nature of implementation and posits that successful implementation of a new innovation (PrEP delivery in FP clinics) will likely require the use of multiple strategies (eg, training, technical assistance, and an internal champion) at multiple levels of the implementation context. The CFIR comprises 39 constructs organized into 5 domains (intervention characteristics, outer setting, inner setting, characteristics of individuals, and process).

### Consent and Institutional Review Board Approval

Given the various types of study participants (patients and clinic staff), phase 2 of this study was divided into two parts for logistical and institutional review board (IRB) purposes. Both parts have been reviewed and approved by the Emory University IRB (IRB# 00107692 and 00111612) and the University of North Carolina at Chapel Hill IRB (IRB# 18-2442 and 19-1784).

Written consent for the clinic staff focus groups, clinic staff web-based surveys, clinic patient surveys, and cohort study will be obtained for all willing participants before participation. Among the web-based survey participants, written consent is asked within the survey. These survey responses are de-identified to protect participants’ privacy. Staff participants indicate interest in a follow-up qualitative interview during the consent process. Verbal consent is obtained over the telephone before the start of the qualitative interview.

### Participants

#### Clinic Staff

Clinic staff participants may include any staff, provider, or administrator employed at any of the three clinics in the phase 2 study. Across all three clinics, at each evaluation time point, there will be up to 100 staff surveys, 45 staff key informant interviews, and 20 staff focus group participants. Cross-sectional enrollment at each evaluation time point rather than a longitudinal assessment of select staff over the entire study period was selected given the staff turnover.

#### Clinic Patients

Patient participants include self-identified, female patients receiving care by a provider on the day of assessment at any of the three clinic sites. As the three clinics see women of varying ages, we will not restrict to younger ages. This will allow for a comprehensive assessment of PrEP implementation in these clinics, across their patient population, and identification of age-specific associated factors. Patient participants who complete the one-time patient exit survey must be aged 13 to 45 years, able to speak and understand English, not self-reported HIV positive, and have completed a visit at one of the three clinics on the day of survey consent. Patient exit surveys will occur approximately once quarterly for up to 9 time points (depending on onset of implementation phase), with up to 60 participants across all three clinics per time point. Patient participants may complete a survey at more than one time point, but their responses will not be linked.

#### Cohort Patients

Patient participants enrolled into the cohort component of this study must meet additional inclusion and exclusion criteria. These participants must have been seen for a patient visit in one of the three implementation clinics during the preceding 60 days and identified as a PrEP candidate based on HIV testing and risk assessment. In addition, they must not be currently enrolled in an HIV vaccine trial, have not been on PrEP for 7 or more consecutive days in the past, not be currently receiving PrEP care outside of the three clinics, and not be currently participating in another PrEP or candidate PrEP study. This cohort has a target enrollment of 300.

### Recruitment

#### Clinic Staff

All clinic staff, including providers and administrators, who are part of FP services at each clinic site will be approached for participation by study staff. Clinic staff will be invited via email to participate in staff surveys. As part of the survey, individuals will be asked if they are interested in potentially participating in key informant interviews. Among those who indicate their willingness, a select group within each clinic will be purposefully selected and invited via email or phone to participate in key informant interviews. Finally, individuals comprising the Clinic Change Team, including the designated PrEP Champion, will be invited via email to participate in focus groups. Clinic leadership at each clinic site will select a PrEP Champion as well as a Clinic Change Team.

#### Clinic Patients

Clinic patient participants will be recruited by trained study staff. Specifically, study staff will attend clinic on days when FP patients are being seen and will review the daily FP clinic schedule with a designated FP clinic staff member to identify individuals to approach for recruitment (see eligibility criteria). Study staff will approach potentially eligible patients and invite them to participate in the exit survey immediately following their provider visit.

#### Cohort Patients

Cohort patient participants will be recruited for the study using flyers and clinic staff referrals. Clinic staff will refer PrEP-eligible women who meet the eligibility criteria and agree to be contacted for a potential research study directly to study staff. In addition, clinic staff will distribute flyers to potential participants with study staff contact information, and study staff will be present in the clinics during regular intervals to facilitate on-site recruitment.

### Incentives

All participants (clinic staff and patients) will be offered compensation for their time.

### Data Collection

#### Clinic Staff

Aggregate of quarterly clinic-level data will be collected via clinic medical chart abstraction over the course of the study.

Quantitative and qualitative data will be collected for staff participants at three time points (approximately annually). Quantitative data will be collected via a web-based survey [31], taking approximately 20-30 min to complete. For the staff survey, the study staff will distribute the survey link via clinic email addresses. Qualitative data collection includes key informant interviews and focus groups. Key informant interviews will last approximately 60 min and will occur either in person or over the telephone. Staff participants who comprise the Clinic Change Team, which includes clinic staff and administrators who participated in PrEP implementation trainings and are involved in PrEP implementation in their clinics, will participate in in-person focus groups. Focus groups are expected to last 60-90 min. Trained study staff will conduct key informant interviews and focus groups, with at least two study staff facilitating focus groups (one as facilitator and one as a note taker). All interviews and focus groups will be audio recorded and sent for professional transcription.

#### Clinic Patients

Clinic patient participants will complete a brief (less than 10 min) interviewer-assisted patient exit survey following a visit at one of the three FP clinics. Clinic patient participants will be recruited approximately quarterly.

#### Cohort Patients

Data relevant to cohort patient participants will encompass medical chart review, interviewer-administered surveys, audio computer-assisted self-interviews, and pharmacologic and laboratory data. Data for cohort patient participants will be collected by trained study staff at the baseline, 3-month, and 6-month follow-up visits, with the allowance for interim visits as needed.

### Quantitative Data Analysis

#### Sample Size

The prospective cohort study will attempt to enroll up to 300 PrEP-eligible women across three clinics. Sample sizes pertaining to clinic staff and clinic patient participants were not chosen to yield a specified level of statistical power for hypothesis testing or to provide a specific level of precision for estimation of a target estimand. However, based on a sample size of 300, the study will provide the ability to estimate the percentage of PrEP-eligible women who are willing to initiate PrEP at baseline within a margin of error of approximately 4.5% (assuming a true uptake percentage of 20%). If instead only 150 participants are enrolled, the margin of error increases to 6.4%. If the actual uptake is lower than assumed, the margin of error will be less than stated.

As this is the first study of its kind focusing on the components of the PrEP cascade in this population and estimates for uptake of PrEP are not available for women seeking care in FP clinics in the Southern United States, the distribution of patients who achieve each step of the PrEP cascade is not well understood. As a result, the quantitative data analysis objectives for the study are not powered for any specific hypothesis testing aim or to achieve a particular level of precision for a specific target estimand. However, based on a sample size of 300, the study will provide 70% power or more to detect a 12% difference in PrEP uptake between race groups (binary) provided the sample size distribution between the two race groups is not more imbalanced than 2:1, assuming a true uptake percentage in two groups of 15% and 27%, and based on a 5% significance level for the test.

#### Outcomes

**Figure 2:**
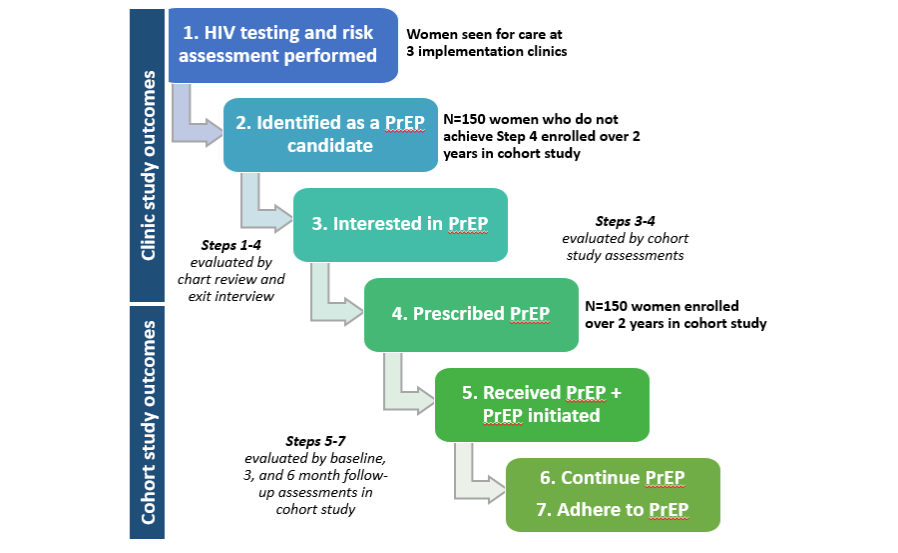
Overview of the study design and outcomes. PrEP: pre-exposure prophylaxis.

The primary objectives of this study are to describe how implementation strategies affect the implementation of PrEP care in FP clinics, by analyzing clinic study data over time from pre- to post-PrEP implementation (Table 1), and to describe the PrEP cascade among women seeking care in FP clinics in Metro Atlanta (Table 2). The outcomes within the PrEP cascade include HIV testing, risk assessment, and prevention counseling inclusive of PrEP; PrEP prescriptions; and PrEP uptake, persistence, and adherence in PrEP-eligible women (Figure 2). As data capturing the entire span of the PrEP cascade and factors impacting successful implementation could not be captured by one data source, multiple data sources were necessary.

**Table 1:** Description of outcomes supporting the study’s primary objective to describe how implementation strategies affect the implementation of HIV pre-exposure prophylaxis care in family planning clinics among women aged 13 to 45 years.

Outcomes		Definitions
**Clinic staff (survey, focus groups, and key informant interviews)**		
	Implementation processes	Degree of adherence to the clinic-specific PrEP^a^ implementation plans over assessment period
	Factors affecting implementation	CFIR^b^-guided factors, assessed after PrEP implementation begins, that contribute to adherence to or deviations from the clinic-specific PrEP implementation plans
**Clinic chart abstraction**		
	HIV testing	Change over time from pre- to post-PrEP implementation in the percentage of visits at the clinic in women aged 13 to 45 years where HIV testing was performed
	HIV risk assessments	Change over time from pre- to post-PrEP implementation in the percentage of visits at the clinic in women aged 13 to 45 years with a documented HIV risk assessment
	PrEP prescriptions	Change over time from pre- to post-PrEP implementation in the percentage of visits at the clinic in women aged 13 to 45 years at which a prescription for PrEP was received
**Clinic patients (exit survey)**		
	HIV prevention counseling	Change over time from pre- to post-PrEP implementation in the percentage of clinic patient participants who report whether they received HIV prevention counseling during their visit to the clinic that day
	HIV prevention counseling inclusive of information about PrEP	Change over time from pre- to post-PrEP implementation in the percentage of clinic patient participants who receive HIV prevention counseling that includes information about PrEP during their visit to the clinic that day

^a^PrEP: pre-exposure prophylaxis.

^b^CFIR: Consolidated Framework for Implementation Research.

**Table 2:** Description of outcomes supporting the study’s primary objective to describe the HIV pre-exposure prophylaxis care cascade among women aged 13 to 45 years.

Outcomes		Definitions
**Clinic chart abstraction**		
	HIV testing	Percentage of visits at the clinic in women aged 13 to 45 years where HIV testing was performed
	HIV risk assessments	Percentage of visits at the clinic in women aged 13 to 45 years with a documented HIV risk assessment
	PrEP^a^ prescriptions	Percentage of visits at the clinic in women aged 13 to 45 years at which a prescription for PrEP was received
**Clinic patients (exit survey)**		
	HIV prevention counseling	Percentage of clinic patient participants who report whether they received HIV prevention counseling during their visit to the clinic that day
	HIV prevention counseling inclusive of information about PrEP	Percentage of clinic patient participants who receive HIV prevention counseling that includes information about PrEP during their visit to the clinic that day
**Cohort patients**		
	PrEP uptake	Participant who receives a PrEP prescription at the baseline visit, fills their prescription, and self-reports initiating PrEP
	PrEP persistence	Participant who attends at least one follow-up visit and has a documented pharmacy refill of PrEP medication at least once during each 3-month interval
	PrEP adherence—hair sample	Average tenofovir concentration measured using a small hair sample (ng/mg); percentage of participants with adherence level consistent with 7 doses per week (≥0.0370 ng/mg) [34,35]
	PrEP adherence—blood sample	Percentage of participants with dried blood spot tenofovir concentration≥1250 fmol/punch
	PrEP adherence—urine sample	Percentage of participants with tenofovir detected by urine immunoassay
	PrEP adherence—self-report	Percentage of participants reporting no missed doses in the past 7 days; percentage of participants reporting very good or excellent adherence (5 or 6 on a 6-level Likert scale) [36] in the past 30 days; percentage of participants who self-report adherence of 90% or higher
	PrEP adherence—pharmacy fill	Percentage of participants with 80% adherence by medication possession ratio defined as the number of dispensed pills divided by the number of days since starting PrEP [37,38]

^a^PrEP: pre-exposure prophylaxis.

The clinic staff survey outcomes include the following CFIR domains to support a secondary objective of exploring provider and staff- and clinic-level factors and their association with steps in the PrEP care cascade. These steps include (1) inner setting readiness for implementation, (2) inner setting: implementation climate, (3) characteristics of individuals: knowledge and beliefs, (4) characteristics of individuals: self-efficacy, (5) characteristics of individuals: attitudes, (6) inner setting: leadership engagement, and (7) inner setting: available resources. These outcomes are more specifically defined in the protocol for phase 1 of this study [37].

Cohort patient outcomes to support a secondary objective specific to individual patient-level factors and their association with steps in the PrEP care cascade include comparing incidence of sexually transmitted infections, pregnancy, and HIV infection; contraception use; and contraception method adherence among the PrEP participants compared with the non-PrEP participants and characterizing PrEP interest, initiation, and indication, over time, for all participants.

#### Statistical Analysis

For quantitative outcomes defined based on chart abstraction (HIV testing, HIV risk assessments, and PrEP prescriptions) and clinic patient exit surveys (HIV prevention counseling, HIV prevention counseling including information about PrEP), we will compute the proportion of patients meeting the outcome definition (eg, portion who received an HIV test) along with 95% confidence intervals. For analyses based on clinic patient exit surveys, data from all exit surveys will be included in the statistical analysis.

For quantitative outcomes defined for cohort patients, all data will be included in analyses for all patients from enrollment until completion of the study or until the patient discontinues participation or becomes lost to follow-up. For the PrEP uptake and PrEP persistence outcomes, the proportion of patients meeting the outcome definition along with 95% confidence intervals will be computed. Association analyses will be performed using mixed logistic regression models to determine whether outcomes are associated with patient-level characteristics such as age, race, education level, and relationship status.

For PrEP adherence outcomes, data from all patients who initiate PrEP at their baseline visit will be included in the analyses. The proportion of patients meeting each PrEP adherence outcome definition along with 95% confidence intervals will be computed at the 3- and 6-month post baseline time points. Analyses will be performed using mixed logistic regression models to determine whether adherence outcomes are associated with patient-level characteristics such as age, race, education level, and relationship status and whether adherence rates change over the 6-month observation period. Analyses will be performed that also incorporate data from patients who initiate PrEP after their baseline visit. Agreement between the different PrEP adherence outcome measures will also be assessed using standard methods (eg, weighted kappa statistic).

Before the analysis of quantitative data, a comprehensive statistical analysis plan will be developed and finalized. Primary and secondary study outcomes and statistical analysis methods will be described in full detail in the study’s statistical analysis plan. This plan will be accessible on ClinicalTrials.gov once available.

### Qualitative Data Analysis

Coding of the key informant interview and focus group data will follow a content analysis and deductive approach, using the CFIR. Analysts will remain open to new themes that may arise inductively from the data. The coding process will follow a consensual research approach, where multiple judges are used throughout the data analysis to ensure multiple perspectives. Then, consensual validation is achieved through a process of deliberation and consensus among judges, and then, an individual *external* to the team (an outside qualitative expert) will review the process to maximize the validity of the findings. After the codebook is finalized, the qualitative coding will be conducted in 3 phases: (1) organize data with codes and build a foundation for case-based analysis (each clinic is considered a case); (2) using NVivo 11 (QSR International), a pair of analysts will code transcripts and meet to reach consensus, and then, final codes will be applied for each transcript; and (3) pairs of analysts will draft a case memo, organized by constructs. The case will be developed iteratively as each transcript is coded, added to, and used to refine the memo. Rigor for qualitative research will be employed by having verbatim transcripts, structured codebook and coding training, double coding, and team consensus on data themes.

### Integration and Dissemination of Findings

For objective one addressing clinic-level factors related to PrEP adoption and implementation, clinic study primary outcomes (Table 2; chart abstraction and clinic patient outcomes), clinic staff survey secondary outcomes (CFIR domains), and data themes from staff interviews and focus group data will be summarized overall and for each of the three FP clinics. For objective two evaluating the effect of PrEP integration in FP clinics on PrEP uptake, persistence, and adherence, each step in the PrEP cascade and its associated factors will be summarized. Findings will be disseminated to key stakeholders, including the three participating FP clinics.

## Results

Research activities for this study began in February 2018 and are ongoing. As of March 08, 2020, all three clinics have begun providing PrEP, and 120 clinic patient exit surveys, 1 focus group with 4 participants, 1 key informant interview, and 13 cohort patient baseline visits have been completed. Qualitative data analysis is scheduled to begin in Fall 2020.

## Discussion

Although FP clinics may be an ideal setting for PrEP delivery, there is a lack of available data from health care providers, administrators, and patients to guide optimal integration of PrEP into various safety net clinical settings, particularly for women’s health care settings [39,40]. By simultaneously evaluating multilevel factors associated with the level of PrEP adoption and implementation and the effects of PrEP implementation on PrEP uptake, persistence, and adherence among women over a 6-month follow-up period, this study will provide an abundance of meaningful data to further guide PrEP integration in Title X–funded clinics across the Southern United States.

## Abbreviations

ATN: Adolescent Medicine Trials Network for HIV/AIDS Interventions
AYAW: adolescent and young adult women
CFIR: Consolidated Framework for Implementation Research
EPIS: Exploration, Preparation, Implementation, and Sustainment
FP: family planning
IRB: institutional review board
PrEP: pre-exposure prophylaxis
